# Systemic protothecosis in an immunocompetent patient

**DOI:** 10.1186/s40794-022-00180-8

**Published:** 2022-10-15

**Authors:** Alvano Trespalacios Sierra, Bárbara Arroyo-Salgado, Jesús Rodriguez-Blanco, Ingrid Tibocha Gordon, María Cristina Martínez-Ávila

**Affiliations:** 1 Intensive Care Unit, Nuevo Hospital Bocagrande, Cartagena, Colombia; 2 Biomedical, Toxicological and Environmental Sciences Research Group (Biotoxam), Universidad de Cartagena, Cartagena, Colombia; 3 Emergency Department, Nuevo Hospital Bocagrande, Cartagena, Colombia; 4 Department of Epidemiology and Public Health, Biomedical, Toxicological and Environmental Sciences Research Group, Cartagena, Colombia

**Keywords:** Emerging diseases, protothecosis, *Prototheca wickerhamii*, algaemia, treatment

## Abstract

**Background:**

Although uncommon, there is increasing interest and public health concerns of the pathogen *Prototheca spp*, a ubiquitous achlorophilic microalgae that can affect both humans and animals. High mortality rates have been reported in immunocompromised patients with disseminated infection, but no data is available in the immunocompetent population.

**Case Presentation:**

We present the case of a 59-year-old man from rural area of Colombia who was admitted to the intensive care unit due to decompensated heart failure that was difficult to medically manage, with development of septic shock and isolation of *Prototheca wickerhamii* from blood culture. Fluconazole and Amphotericin B were given with successful outcome.

**Conclusions:**

To date, protothecosis and its virulence factors and pathogenesis remain to be fully understood, in our case the isolation of this microalga and its implication of exacerbating chronic conditions such as heart failure is unclear. The medical-scientific community is invited to study this microorganism to determine effective management strategies, as well as its timely identification, treatment, and control, to avoid fatal outcomes.

## Background

Infection due to *Prototheca* spp., a ubiquitous saprophytic achlorophilic microalgae is emerging in some clinical contexts and is considered a potential threat to public health [1]. This microorganism has been found to colonize skin, nails, respiratory and digestive tracts of humans and animals [2, 3]. *Prototheca* is also found in the environment reservoirs as well (e.g., slime flux of trees, grass, wastewater) [2, 3].

The importance of *Prototheca spp* lies in the sudden appearance and capacity of infection in immunocompetent and immunosuppressed individuals [2]. Although pathogenesis remains unclear, most cases of reported protothecosis were in immunocompromised patients. Few have been reported in immunocompetent individuals. The route of infection is still unknown, this along with the lack of clinical and diagnostic suspicion of protothecosis can lead to delays in appropriate diagnosis and management of this infection.

To date, few effective therapies are established, with no guidelines on performing susceptibility testing, its interpretation, and issues with quality control of in vitro susceptibility testing (i.e., MICs not always reproducible) [2, 3]. Management of protothecosis is also controversial with varying treatment responses with different regimens [3].

Herein, a clinical case is presented of an immunocompetent patient who was admitted to the intensive care unit (ICU) due to shock of unknown etiology with blood culture isolation of *Prototheca wickerhamii*.

## Case presentation

A 59-year-old man, farmer, living in a rural area of Cartagena, ​​Colombia (elevation 17 m / 55.77 ft above sea level) was initially admitted to a peripheral hospital for decompensated heart failure. He has a history of hypertension, well-controlled type 2 diabetes mellitus (last HbA1c 2 months before admission was 6.6%), and congestive heart failure with reduced ejection fraction (LVEF 30%). At home, he takes carvedilol, spironolactone, furosemide, aspirin, and metformin.

Vitals at the other institution were unremarkable but had signs and symptoms of decompensated heart failure (dyspnea, fatigue, pitting edema, wheezing). Post-admission day 20, he was transferred to our ICU for hemodynamic monitoring due to high risk of shock and complications. On admission, his blood pressure was 106/84 mmHg, heart rate 116 beats/minute, 75% on room air returning to 96% with high-flow nasal cannula at 6 L/minute.

On physical examination he was afebrile, had no skin lesions, toxic appearance, or signs of systemic inflammatory response. He had jugular engorgement, normal cardiopulmonary auscultation, and Grade + 2 pitting edema of lower limbs. Chest x-ray showed signs of pulmonary congestion. Echocardiogram revealed a dilated heart disease, left ventricle with severe global systolic dysfunction, depressed ejection fraction 18%, severe biatrial dilatation and moderate mitral valve regurgitation without thrombi or vegetations.

Laboratory tests revealed acute kidney injury and mild metabolic acidosis (Table 1). His management was continuing home medications and adding angiotensin II receptor blockers (ARB) and intravenous loop diuretic.

**Table 1:** Laboratory tests of interest during ICU stay

Laboratories	***Admission***	***Day 5***	***Discharge***	***Reference ranges***
Hemoglobin	12	13.5	12.4	12 - 15 mg/dL
Hematocrit	41	42	40	36.0–46.0%
White blood cell count	10,346	25,000	8750	4000–11,000 mm3/L
Neutrophils	50	90	45	55–65%
Lymphocytes	29	8	37	23–25%
Platelet count	297,000	184,000	200,000	150,000–400,000 mm3/L
Serum creatinine	2	2.4	1.94	0.51–0.95 mg/dl
Blood urea nitrogen	51	68	39	12-26 mg/dl
Serum alanine aminotransferase	35	140	30	0–32 U/L
Serum Aspartate Aminotransferase	24	85	25	0–33 U/L
Sodium	137	142	140	135 - 145 mEq/L
Potassium	3.8	5.4	4.2	3.5 - 5 mEq/L
Chlorine	100	98	97	95 - 110 mEq/L
HIV		Negative		Negative
VDRL		Negative		Negative
C3		96		80 - 200 mg/dL
C4		50		10 - 70 mg/dL
Hepatotrophic viruses (AgsHB, AntiHB, HCV)		non-reactive		non-reactive
C-reactive protein		28		< 1 mg/dl
Partial thromboplastin time	30	28	3. 4	25 to 35 seconds
Prothrombin time	13.9	14	13.8	11 to 15 seconds
Total bilirubin		1.1		0.1 - 1 mg/dL
Direct bilirubin		0.87		< 0.4 mg/dL
Indirect bilirubin		0.24		< 0.6 mg/dL
TSH		2.51		0.37–4.7mIU/L
FT4		1.03		0.4–1.7 ng/dL
Arterial blood gases				
pH	7.34	7.28	7.39	7.35–7.45
PCo2	31	40	45	30–45
PO2	96	88	92	80–100
HCO3	13.6	11	18	20 ± 2
PaO2/FiO2 ratio	460	375	430	> 300

The patient presented a torpid evolution with the development of a febrile illness (temperature 38.6 °C) 3 days after admission to the ICU (23 days of hospitalization), associated with sustained ventricular tachycardia reaching values of 198 beats/minute and sustained hypotension with mean arterial pressure 63-66 mmHg. He was prescribed digoxin without improvement, followed by amiodarone infusion and full anticoagulation. Labs revealed severe leukocytosis with neutrophil predominance, and mild hepatitis.

Suspecting a possible infectious focus that decompensated the patient, septic workup was initiated and a carbapenem and antifungal (Fluconazole) were started given risk factors (comorbidities and prolonged hospitalization in another institution). Finally, blood cultures in two sets at 120 hours of incubation revealed the presence of achlorophyllic algae, confirmed as *Prototheca wickerhamii* by MALDI-TOF system. No other evidence of disseminated infection was documented. In this patient, the suspected source of infection was his home surroundings and occupation (farmer living in a rural area) along with risk factors such as diabetes.

The patient was assessed by Infectious Diseases who recommended fluconazole 400 mg IV/day and repeat blood cultures. Repeat blood cultures taken at 96 hours after initiating antifungals showed persistence of the microorganism, treatment was changed to liposomal Amphotericin B (5 mg/kg IV/day) for 14 days. Subsequent repeat blood cultures documented clearance of the algaemia post-treatment. Once the treatment was completed, he presented clinical and biochemical improvement, allowing hospital discharge.

In outpatient follow-up after discharge from hospital, he remains well off antifungals and asymptomatic.

## Discussion

Regarding human protothecosis, more than 200 cases of infection by *Prototheca spp* were reported in the literature [2]. Clinical manifestations include cutaneous forms (66%), olecranon bursitis (15%) and disseminated or systemic infections (19%) [2]. No direct human-to-human transmission has been reported, but infection through contact (such as by traumatic inoculation or insect bites) with the algae from potential sources have been reported [3, 4]. Possible sources of *Prototheca spp* include rural areas, trees, grass, soil, sewage, animals (cattle, deer, and dogs), animal droppings, foods (e.g., butter, potato skins, bananas, cow’s milk) [3].

Disseminated infection and those with risk factors such as immunosuppression or oncological patients are associated with higher mortality rates, a mortality of 56% has been reported [2]. In immunocompetent patients there are still no data, nevertheless there are certain occupations that are considered at higher risk of exposure to the pathogen such as farmers, workers of rice paddies and fishermen [2, 3]. In our case, it was impossible to know if the microorganism was acquired before or during the hospital stay. Nosocomial infections have been reported, though primarily associated following orthopedic or surgical procedures [3], even though our patient did not receive any invasive procedures when he developed symptoms during his hospitalization. But we considered he was colonized before, and heart failure decompensation favored the systemic manifestation symptoms.

Virulence factors for protothecosis are unknown. Our patient with history of diabetes (reported as a potential risk factor for protothecosis) developed severe algaemia with decompensated heart failure leading to a critical state in the ICU. It is suggested that the presence of comorbidities (e.g. cancer, diabetes, steroid use, AIDS, alcoholism, dialysis, surgery) is a risk factor that facilitates the infectious condition, although it is still unknown if they determine the severity of the infection [3].

Identifying *Prototheca* spp. often requires both microbiological and histopathological testing performed by trained personnel, as relying on only one method may result in misidentification for other similar-appearing fungal pathogens [2, 3]. For microbiology, this may involve using automated microbiological analyzes (blood cultures), with the help of Sabouraud dextrose agar, *Candida spp* chromogenic agar, biochemical carbohydrate assimilation tests, immunofluorescence, and molecular analyses [5]. For histopathology, tissue biopsies can be prepared with periodic acid-Schiff (PAS), Hematoxylin and Eosin or Grocott-Gomori methenamine silver (GMS) staining, which may show a characteristic morula with a thick wall and internal septations, with associated chronic granulomatous inflammation and infiltration of histiocytes, lymphocytes, and giant cells [5]. It is worth to highlight the need for diagnostic suspicion to aid in identification of the organism microbiologically.

To date there is no defined standard treatment for this infection, antifungals (e.g, amphotericin B, itraconazole, fluconazole, voriconazole) and antibacterials (e.g, tetracycline, gentamicin, amikacin) have been used with variable success rates [6–11]. In Colombia, 10 cases of protothecosis shown in Table 2 have been described, this being the first in critical condition with hematogenous dissemination. All reported patients had favorable outcomes despite not having a standardized treatment and receiving different management strategies. There are no data indicating whether there is a condition differentiated by sex, although to date it seems that in Colombia it is more frequent in men.

**Table 2:** Reported cases of Protothecosis in Colombia from 1970 to 2021

First Author/Reference	Age, Sex	Geographic location	Species	Type of infection	Site of infection	Diagnostic procedures	Risk factors	Treatment	Outcome	Year
Linares G [6]	No information	San Andrés	*Pw*	Cutaneous	Left lower limb	Histopathology	Not reported	Surgical: Removal	Cure	1970
Guzman M [7]	40 M	Medellin	*P.*spp	Musculoskeletal	Olecranon: bursitis	Blood Culture	Not reported	None	Cure	1983
Guzman M [7]	32F	Tai-Pei, consulted in Bogotá	*P.*spp	Cutaneous	Nail: right hand	Histopathology	Not reported	Imidazole	Cure	1983
Guzman M [7]	48 M	Bogotá	*P.*spp	Musculoskeletal	Olecranon: bursitis	Histopathology and blood Culture	Not reported	Local heat	Cure	1983
Linares G [6]	50 M	Cali	*Pw*	Cutaneous	Right elbow	Histopathology	Steroid use	Ketoconazole	Cure	1992
Rodriguez G [8]	43 M	Bogotá	*Pw*	Cutaneous	Nasal Dorsum	Histopathology	Intravenous drug use	Surgical: Removal	Cure	2001
Castro LA [9]	72F	Cali	*P.*spp	Cutaneous	Neck, lower limbs	Histopathology	Not reported	No information	No information	2014
Ramirez I [10]	47 M	Medellin	*Pw*	Musculoskeletal	Olecranon: bursitis	Histopathology and Blood Culture	Liver kidney transplant	Surgical: Excision + Amphotericin B	Cure	2015
Velez-Mejia C [11]	43 M	Medellin	*Pw*	Cutaneous	Left lower limb	Histopathology and Blood Culture	Steroid use	Amphotericin B	Cure	2017
Current Case	59 M	Cartagena	*Pw*	Hematogenous	Algaemia	Blood Culture	Diabetes	Amphotericin B	Cure	2021

*Pw Prototheca wickerhamii, P.spp Prototheca spp, M* Male, *F* Female

## Conclusion

In conclusion, systemic protothecosis has so far been surrounded by an aura of uncertainty. It is very likely that the infection is more frequent than we think, considering the difficulties in isolating and diagnosing it. As the reports of this disease progress, we will have more solid evidence on its epidemiological profile, cross-reaction with other microorganisms, standard preventive, and therapeutic measures for *Prototheca spp* in order to avoid fatal outcomes, particularly in patients with chronic diseases or states of immunosuppression.

## Data Availability

We have presented the data of the patients in the manuscript as tables and have submitted the figures separately as figures.

## References

[1] Zhao F, Chen M, Fu Y (2020). Multiple cutaneous infections caused by Prototheca wickerhamii. J Clin Lab Anal.

[2] Todd JR, Matsumoto T, Ueno R, Murugaiyan J, Britten A, King JW, Odaka Y, Oberle A, Weise C, Roesler U, Pore RS (2018). Medical phycology 2017. Med Mycol.

[3] Lass-Flörl C, Mayr A (2007). Human protothecosis. Clin Microbiol Rev.

[4] Rao PV, Sethuraman N, Ramanathan Y, Gopalakrishnan R (2018). Disseminated Protothecosis Caused by*Prototheca zopfii*in a Liver Transplant Recipient. J Glob Infect Dis.

[5] Kano R (2020). Emergence of fungal-like organisms: prototheca. Mycopathology.

[6] Linares G, Baker RD (1970). Cutaneous protothecosis: Infection caused by algae. Arch Col Med El Salvador.

[7] Guzman M, Ramirez G, Buitrago B (1983). Protothecosis: report of three cases. Biomedical..

[8] Rodríguez G, Ordonez N (2001). Make your diagnosis. Biomedical.

[9] Castro LA, Alvarez MI, Ramirez MT (2014). Cutaneous protothecosis: presentation of a case with emphasis on histopathology. IATREIA.

[10] Ramírez I, Nieto-Ríos JF, Ocampo-Kohn C, Aristizábal-Alzate A, Zuluaga-Valencia G, Muñoz Maya O, Pérez JC (2016). Protothecal bursitis after simultaneous kidney/liver transplantation: a case report and review. Transpl Infect Dis.

[11] Velez-Mejia C, Velez-Londoño J (2017). Prototheca: a danger underwater. IDCases.

